# Sociodemographic factors have very little influence on adherence to topical drugs in patients with psoriasis

**DOI:** 10.1016/j.jdin.2024.07.013

**Published:** 2024-08-27

**Authors:** Mathias Tiedemann Svendsen, Klaus Ejner Andersen, Steven R. Feldman, Sören Möller, Line Planck Kongstad, Anna Mejldal

**Affiliations:** a Department of Clinical Research, University of Southern Denmark, Odense, Denmark; b Department of Dermatology and Allergy Centre, Odense University Hospital, Odense, Denmark; c Department of Dermatology, Wake Forest University School of Medicine, Winston-Salem, North Carolina; d Open Patient Data Explorative Network (OPEN), Odense University Hospital, Odense, Denmark; e Danish Centre for Health Economics (DaCHE), University of Southern Denmark, Odense, Denmark

**Keywords:** adherence, clinical trial, psoriasis, sociodemographic factors

*To the Editor:*

Adherence to psoriasis treatment with topical drugs is low, because of various barriers, including disease and treatment-related factors. Sociodemographic factors may also contribute.^1^ However, in a randomized controlled trial investigating the use of calcipotriol and betamethasone dipropionate foam in 122 Danish patients with psoriasis, sociodemographic factors had little influence on patients’ adherence to the topical therapy.^2^

To further investigate the effect of sociodemographic factors on adherence, we applied a *post hoc* analysis of another randomized controlled trial including 103 Danish patients with mild-to-severe psoriasis.^3^ The primary objective of the trial was to investigate whether regular support provided by specialist dermatology nurses over a 48 week-period could improve adherence to topical drugs. Three adherence measurements were obtained at the last study visit week 48: primary adherence, that is, adherence measured as the proportion of patients who filled a new prescription, the weight of medication used, and self-reported adherence. In this letter, we describe one of the secondary outcomes of the study, the relationship between sociodemographic factors and adherence to treatment.

We investigated the dichotomized adherence measures (with patients considered adherent according to an adherence-rate cutoff of 80%), with respect to the same sociodemographic factors, using bivariate and multivariate logistic regression on complete cases (92 cases).

Bivariate and multivariate analyses gave comparable results. There were no associations between adherence and most sociodemographic factors (Fig 1). However, there was a negative association between having a further education and adherence only when assessed by primary adherence (odds ratio [OR], 0.18; 95% CI, 0.042-0.80; *P* = .02), whereas age >50 years had a negative association on adherence when assessed by weight of applied medication (OR, 0.31; 95% CI, 0.099-0.95; *P* = .04). On the other hand, there was a positive association between being a woman (vs man) and adherence only when assessed by weight (OR, 2.81; 95% CI, 0.95-1.08; *P* = .04). There was also a positive association between being a smoker and reporting good adherence (OR, 3.94; 95% CI, 1.28-12.08; *P* = .02).

**Fig 1:**
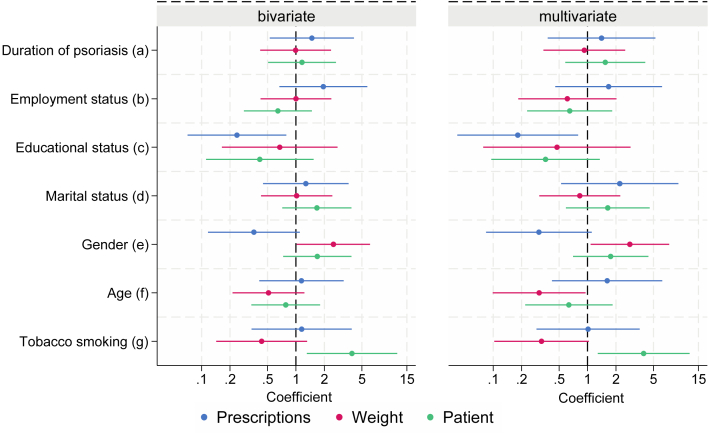
The association between sociodemographic factors and adherence to treatment with topical corticosteroids with or without calcipotriol. We assessed dichotomized adherence measures (adherence-rate cutoff of 80%), with respect to sociodemographic factors, using bivariate and multivariate logistic regression. Regression coefficients CI including 1 shows nonsignificant associations. A coefficient with CI >1 indicates the sociodemographic factor is associated with better adherence, whereas a coefficient with CI <1 indicates the sociodemographic factor is associated with worse adherence. There were only minimal nonconclusive differences between bivariate and multivariate analyses. Nearly all regression coefficients showed nonsignificant associations (ie, coefficients around 1) between sociodemographic factors and adherence to topical treatment. Prescription refers to primary adherence, calculated as patients filling at least one prescription at the pharmacy. Weight refers to adherence assessed by the weight of topical drugs used. Patient refers to self-reported adherence. ^a^Duration > 20 years, ^b^not full-time employed, ^c^further education, ^d^unmarried or divorced, ^e^female, ^f^age > 50 years, ^g^smoker.

The modest, statistically significant correlations may be related to a variety of factors. People with more education might be more skeptical about the use of corticosteroids.^4^ Older patients may find it more bothersome to apply the topical drugs. The tendency to overreport self-reported adherence could contribute.

Some groups, including women, have been reported to be more adherent to antipsoriatic drugs.^5^ This finding has since been repeatedly recited for decades and it may tend to have become a general conception that women are more adherent to prescribed antipsoriatic drugs.

If health care professionals continue assuming some sociodemographic groups of patients are more adherent, it could lead to less attention to assuring adherence in those groups and potentially poorer outcomes.

This study is limited by the small sample size, not considering unmeasured confounding, such as ethnicity.

In conclusion, adherence to topical treatment is a challenge in most patients with psoriasis. Dermatologists, their health care personnel, and the health care system in general could perhaps focus more on improving adherence in all topically treated patients, perhaps by supporting patients on a regular, long-term basis.^3^

## Conflicts of interest

Drs Svendsen and Andersen received a grant from the LEO Foundation to conduct the trial. Dr Feldman is a speaker for AbbVie, Alvotech, Amgen, BMS, Janssen, Lilly, Regeneron, Sanofi, and Sun, is a consultant for AbbVie, Accordant, Almirall, Alvotech, Arcutis, Arena, Argenx, Biocon, BMS, Boehringer, Dermavant, Forte, Helsin, Janssen, LEO Pharma, Micreos, Mylan, Novartis, Ono, Pfizer, Samsung, Sanofi, Sun, UCB, vTv, and Voluntis, conducts research for AbbVie, Almirall, BMS, Galderma, Janssen, Lilly, Novartis, Pfizer, and UCB, and holds stocks in Sensal Health. Drs Möller, Kongstad, and Mejldal have no conflicts of interest to declare.
